# Emergence of Variability in Isogenic *Escherichia coli* Populations Infected by a Filamentous Virus

**DOI:** 10.1371/journal.pone.0011823

**Published:** 2010-07-27

**Authors:** Marianne De Paepe, Silvia De Monte, Lydia Robert, Ariel B. Lindner, François Taddei

**Affiliations:** 1 INSERM, Unité 1001, Génétique Moléculaire Evolutive et Médicale, Paris, France; Université Paris-Descartes, Paris, France; 2 Ecole Normale Supérieure, UMR 7625, Ecologie et Evolution, Paris, France; Université Pierre et Marie Curie-Paris 6, UMR 7625, Ecologie et Evolution Paris, France; CNRS, UMR 7625, Ecologie et Evolution, Paris, France; 3 AgroParisTech ENGREF, Paris, France; Albert Einstein College of Medicine, United States of America

## Abstract

The spread of epidemics not only depends on the average number of parasites produced per host, but also on the existence of highly infectious individuals. It is widely accepted that infectiousness depends on genetic and environmental determinants. However, even in clonal populations of host and viruses growing in homogeneous conditions, high variability can exist. Here we show that *Escherichia coli* cells commonly display high differentials in viral burst size, and address the kinetics of emergence of such variability with the non-lytic filamentous virus M13. By single-cell imaging of a virally-encoded fluorescent reporter, we monitor the viral charge distribution in infected bacterial populations at different time following infection. A mathematical model assuming autocatalytic virus replication and inheritance of bacterial growth rates quantitatively reproduces the experimental distributions, demonstrating that deterministic amplification of small host inhomogeneities is a mechanism sufficient to explain large and highly skewed distributions. This mechanism of amplification is general and may occur whenever a parasite has an initial phase of exponential growth within its host. Moreover, it naturally reproduces the shift towards higher virulence when the host is experimenting poor conditions, as observed commonly in host-parasite systems.

## Introduction

In the development of epidemics it is not only the average parasite production that matters, but also the distribution of secondary cases infected by single individuals. The spread of a disease can be deeply affected by heterogeneities in infectiousness, with high viral charge ‘superspreader’ individuals triggering stronger epidemic events [1]. For better data interpretation and disease control, it is therefore of primary importance to understand how the distribution of virus production is shaped by the underlying host-parasite interactions.

Variability in the outcome of infection has been largely documented and is generally assumed to be due to genetic or environmental variability [2]–[3]. Nevertheless, strong differences in virus production exist even among cells of a clonal bacterial population growing in a homogeneous environment. Already more than sixty years ago, Max Delbrück demonstrated that for the bacterial virus α, cell-cell variation can be extremely larger for burst size than for other physiological parameters like cell size or growth rate [4]. More recently, several studies pointed out the existence of nongenetic large differences between clonal subpopulations in the response to stresses [5]–[6] or more specifically following viral infection [7]–[8]. The mechanisms behind the origin and maintenance of such large differentials are however still unknown.

Here, we focus on the first stages of a viral infection and examine the evolution of viral charge distribution as large cell-cell differences emerge. We performed experiments with *Escherichia coli* clonal populations infected by bacterial viruses, and in particular by the filamentous phage M13. *E. coli* and M13 constitute a model system for the study of host-parasite interaction in non-acute diseases, as filamentous phage establish a chronic infection during which phage particles (virions) exit the cell without cellular lysis. The infection doesn't prevent bacterial growth and division, and the intracellular forms of the virus are shared at division between the two daughter cells. Though this bacterial virus has been extensively studied over the last decades, the dynamics of the epidemics it generates has been only poorly investigated at the individual cell level [9]. The M13 virion consists of a single-stranded DNA (ssDNA) genome surrounded by a protein coating in the form of a long filament. Upon entering the cell, the ssDNA genome is converted into a double-stranded form (dsDNA). This dsDNA form is concurrently transcribed and replicated, giving rise to single-stranded virions that are subsequently extruded from the cell (see [10] and Figure S1 in the Supplementary Informations for details). The synthesis of the F pilus, the receptor of the phage, is blocked during infection, preventing superinfection [10].

We combined single-cell measures and a mathematical model to investigate the mechanisms generating variability in clonal populations of virally infected bacteria growing in a homogeneous environment. By using a modified virus that constitutively expresses a fluorescent protein, we were able to quantify the amount of virus produced per cell and to follow the evolution of the viral charge distribution in the course of infection. We explain the variability in virus accumulation within cells as the amplification of initial differences in bacterial growth rate. A mathematical model shows that this simple mechanism allows us to reproduce the kinetics of the variability and the total virus production in different controlled environmental conditions.

## Results

### Strong variability in phage production commonly occurs

**Figure 1:**
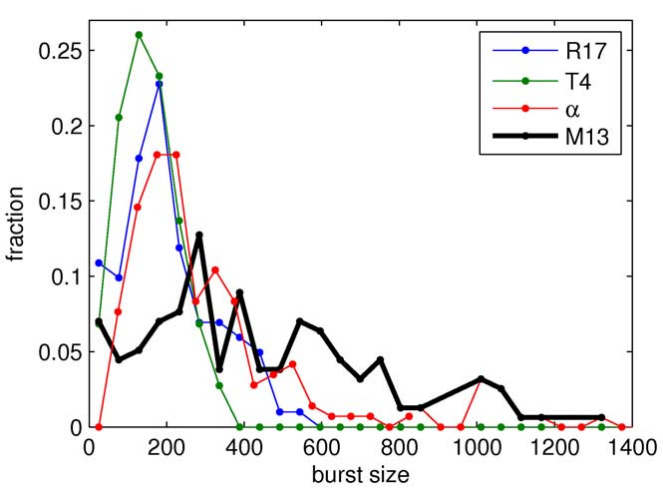
Viral production distribution on single cells of 4 different phages. Distribution of Plaque Forming Units counts from single cell (or lineages in the case of M13) for the following phages: phage α (reproduced from Delbrück, 1945); the double-stranded RNA phage R17, the double-stranded DNA phage T4 and the filamentous single-stranded DNA phage M13. The phage M13 is not lytic, so the number of virions is measured one hour after infection (1–2 host cells). All phages show a strong heterogeneity in the number of virions produced per cell: all the distributions are wide and asymmetric, the extreme case being the filamentous phage M13. See Methods for the culture conditions.

We found that, far from being an unusual property of the phage α studied by Max Delbrück, the high heterogeneity in virus production per cell is a common characteristic of several phages, in spite of marked differences in their infection strategy and impact on the host. Figure 1 shows the distribution of single-cell burst size, measured by counting Plaque Forming Units (PFU, see Methods), for four different bacterial viruses. For all the four viruses studied, PFU distributions have a larger coefficient of variation than any other phenotypic trait measured in a bacterial population, as cell size [4], [8] or cell elongation rate [11]. Coefficients of variations are 0.47 for the phage T4, 0.55 for the phage α (as recalculated from [4]), 0.62 for the phage R17 and 0.77 for the phage M13. Among them, chronic infectious filamentous phage M13 thus displays the widest distribution. We therefore decided to focus on this virus, addressing the questions of the origin of this variability and of its relation with the life style of such a chronic virus.

We expect the contribution of mutations to be minor in the generation of burst size variability. Phage M13 relatively low replication error rate –a maximum of 7.10^−7^ errors per base measured for phage M13 [12]- implies that the genetic variation within viral populations is very small at the time and population scale of our experiments.

### Construction of a fluorescent reporter for measuring single-cell virus production

**Figure 2:**
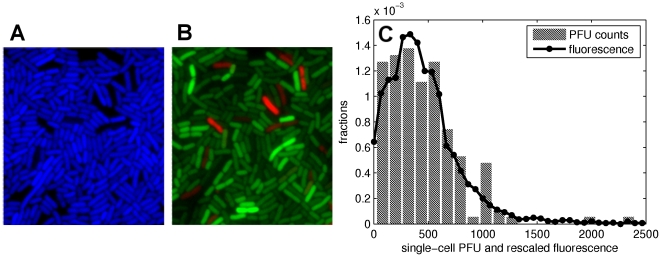
Fluorescence monitors phage production. (**A**) and (**B**): snapshots of bacteria growing in the S condition, plated three hours after infection by M13yfp; (**A**) Cyan fluorescence, measuring the activity of the constitutive promoter p2*rrnB*; (**B**) Yellow fluorescence of the phage-encoded reporter (shown in green for better visualization) and dead cells marked with Propidium Iodide (red). It is immediately evident that cell-cell variability of cyan fluorescence is much smaller than that of yellow fluorescence. While the first measures the fluctuations in promoter activity, the second reflects the number of virions produced per cell. (**C**) The use of yellow fluorescence as a measure of the number of virions produced per cell lineage is validated by the excellent matching between single-cell PFUs distribution and fluorescence distribution one hour after infection and by the strong correlation between total PFU counts and total fluorescence, shown in Fig. S3 in the SI.

The estimation of phage production in single cells by PFU counts is very time-consuming and it is not suited for large sample sizes, nor for high time resolution. In order to quantify the distribution of the viral charge per cell and its kinetics, we hence introduced the Yellow Fluorescent Protein (*yfp*) gene in the phage genome, under the control of the constitutive bacterial promoter p2*rrnB* (see Methods). The gene insertion did not modify the effect of infection on the host (see Figure S2). As a control of noise in promoter expression, we introduced in the bacterial genome the Cyan Fluorescent Protein (*cfp*) gene under the same p2*rrnB* promoter. Figure 2A–B shows typical snapshots of the population that allowed us to quantify the amount of fluorescence, the background expression of the promoter and the proportion of dead cells. The total fluorescence of single cells was normalized by their size, as measured by the number of pixels of the phase contrast image of the cell, and in the following we will refer to it as to “fluorescence intensity”.

On the contrary to viral proteins, YFP are not extruded from the cell and instead accumulate within the cells. The instantaneous YFP production of a single cell is proportional to the number of dsDNA molecules present inside this cell. Similarly, the different elements composing the virions, namely the capsid proteins and the ssDNA are also produced from the dsDNA and therefore virions production is expected to be proportional to the production of YFP. Assuming that binomial partitioning of molecules at cell division does not significantly decorrelates the number of YFP and the number of virions, YFP intensity of a single cell should hence reflect its viral charge.

This statement was not directly demonstrated because of the difficulty to simultaneously measure on individual cells both the number of virions produced and cell fluorescence. Therefore, we first indirectly verified that fluorescence is a good measure of virions production in whole-population assay. We showed that the distribution of PFU counts in single cells one hour after infection rescales to the fluorescence intensity distribution (Fig. 2C). Then, we observed that the total fluorescence intensity of a population (cumulated over all cells) is proportional to the number of virions produced in the same population, as measured by total PFU counts (Fig. S3). Taken together, these results validate the use of YFP fluorescence intensity to monitor the single-cell virion production.

### Kinetics of the fluorescence distribution

**Figure 3:**
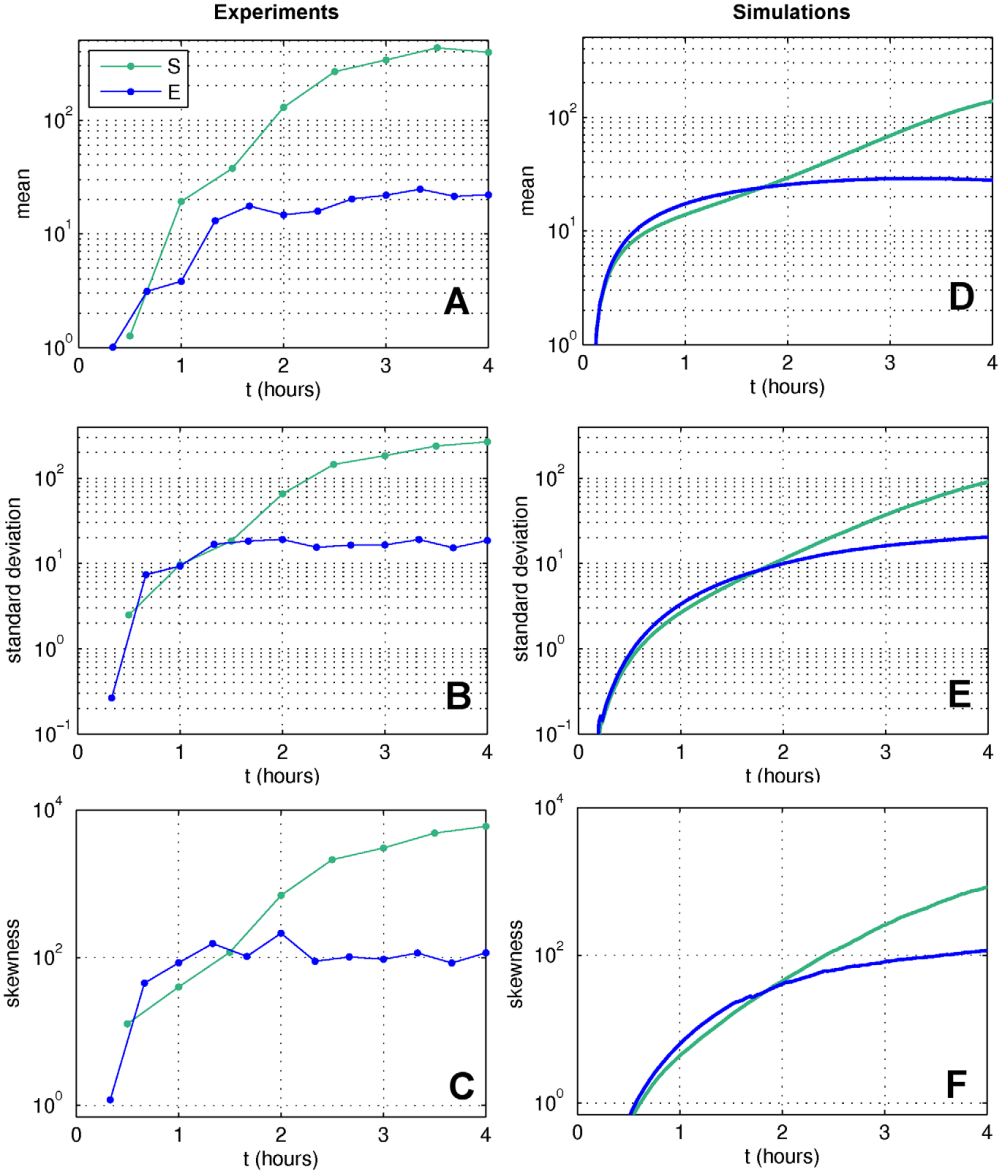
Statistics of the measured fluorescence distribution. First column: (**A–C**) representative dynamics of the first three moments of the fluorescence distribution measured in conditions S (growth in depleting medium, green) and E (culture regularly diluted in rich medium, blue): (**A**) mean, (**B**) standard deviation and **c** skewness. Each distribution is obtained from more than 10,000 infected cells. Second column: (**D–F**) same statistics for the simulations of the model (presented in the Box) describing autocatalytic amplification of initial growth rate differences: (**D**) mean, (**E**) standard deviation, (**F**) skewness. The parameters of the model for intracellular virus dynamics are (see Box): K_d_ = 30, δ = 1.8, τ = 30, f_thresh_ = 10^4^. The two different growth conditions differ by their growth rates: in E condition g_0_ = 1.5 (measured g_0_ = 1.5±.6), in S condition the system was forced with the slope of a fit (2^rd^ order polynomial) of the measured optical density in the course of the non-diluted growth. A delay of 5 minutes has been introduced for accounting for the maturation time of the fluorescent proteins. The initial growth rate distribution is obtained by rescaling the growth rate distribution measured in non-infected cells so that the average growth rate matches that of the infected culture (standard deviation σ = 0.2).

We addressed the dynamics of the emergence of variability among infected cells by following over time cells fluorescence within infected populations. After synchronous infection of the bacteria, we let the population of infected cells grow until they enter the stationary phase. We will call this growth condition S, as saturating. Besides single-cell measurements, traditional population-level quantities have been observed: the absorbance of the culture (Optical Density) and the number of infectious free phage in suspension (PFU). These measures have been repeatedly performed in the course of the infection by the phage, so as to obtain the evolution of the distribution of fluorescence and of the total virus production in the course of time (see Methods). After infection, the mean fluorescence intensity increases rapidly and then at a slower rate as the stationary phase is approached (Fig. 3A). At the same time, standard deviation and skewness of the distribution increase dramatically (Fig. 3B,3C), giving eventually rise to a broad and highly asymmetric distribution, and mortality increases to up to 20%. Fluorescence intensity distribution rapidly attains a coefficient of variation of 80%, much larger than those for growth rate and promoter expression, that are of the order of 10% (see Fig. S4 in the SI for the measured distributions of these two physiological parameters and [13]. This suggests that ‘superspreader’ cells are largely overrepresented with respect to a Gaussian distribution around the same average burst size.

We wondered what is the origin of such variability, and whether its rapid increase at the entry of stationary phase is a response to bacterial density. In order to address these questions, we repeated the experiment and kept the culture in exponential growth (E condition) without nutrient limitations. We did so by re-diluting the culture in fresh medium every hour. We used two different media: rich medium Luria-Bertani (LB) and exhausted medium (supernatant from a two-hours culture) supplemented with yeast extract, the main nutritional component of LB medium. This second medium could contain chemical cues or quorum sensing effectors that carry information on population density in the early stationary phase, but it is not depleted in nutrients. This way, we checked whether variability emerges also if resources are not limiting or it is triggered by signalling processes taking place at the entry in the stationary phase. The culture grown in replenished supernatant behaved as the culture growing in LB medium. This demonstrates that signals of population density have no effect on the dynamics of the fluorescence intensity distribution.

**Figure 4:**
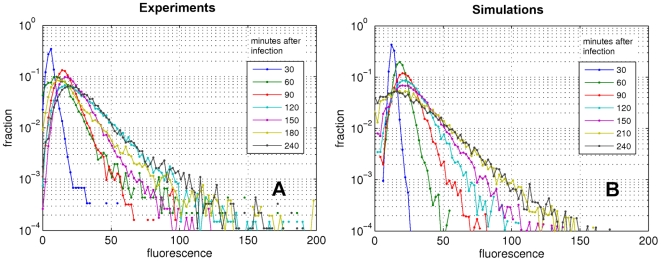
Evolution of the fluorescence distribution with time. (**A**) representative evolution of the repartition of fluorescence intensity at different time points following infection. Each distribution is obtained from more than 10,000 infected cells. (**B**) simulated time evolution of the fluorescence intensity (model parameters as in the caption of Fig. 3). The simulated distribution kinetics results in highly skewed distributions rapidly generated in the first stages of the epidemic, as observed experimentally.

In the E condition, although the nutrient availability is kept almost constant, we still observe (Fig. 3A–C) a drastic increase in variability and skewness of the cellular fluorescence intensity, leading to coefficients of variation comparable to those obtained in the S condition, and almost an order of magnitude higher than other phenotypic traits. This is accompanied however by a much slower increase in average fluorescence. Figure 4A shows the fluorescence distribution in a representative experiment in E conditions: the distribution expands gradually over time and attains a quasi-stationary state a couple of hours after the infection. At each time point the fluorescence repartition is well fitted by a log-normal distribution. In this condition, lower mortality is observed in the population (between 3 and 8% of dead cells).

### A model for the fluorescence distribution dynamics

The experimental observations in saturating growth conditions suggest that viral proteins accumulation may be related to bacterial growth rate, higher fluorescence being associated to slower growth of the culture. That stochastic variations in growth rate can shape protein concentration distribution in cells has been proposed in the context of chromosomally encoded genes [14]. We propose a mathematical model (see Box) to explain the effect of variations in bacterial growth rate on the viral protein concentration distribution. The model describes within-cell viral dynamics as a deterministic outcome of viral replication and dilution due to bacterial growth: viral DNA undergoes a logistic growth and in the meantime is trancribed, producing fluorescent proteins. These proteins accumulate within the cell and, contrary to the virions, are not extruded. We show that the kinetics of the fluorescence distribution in exponential and saturating growth can be mechanistically explained on the basis of two features: a) a heritable (epigenetic) growth rate difference between the virus and its host, b) the autocatalytic reproduction of virus. At the beginning, cells are assigned a growth rate drawn from the growth rate distribution of non-infected bacteria (Fig. S4) rescaled according to the measured average growth rate of infected cultures. Then, cells of the same lineage remain in the same class of growth rate, consistent with the observation that in plated microcolonies of infected bacteria larger colonies had lower fluorescence, and differences within colonies were smaller than among colonies. This assumption is also comforted by the fact that a correlation of 0.5 (Spearman's rank correlation, P-value<10^−16^) was evidenced in the growth rate of mothers and daughters *E. coli* cells [15], but is clearly an extreme simplification aimed at evidencing the effect of cell history on the variability buildup, as pointed out later in the discussion. The parameters of the model quantifying viral replication and virus-induced cell mortality were set identical in every cell and constant in time.

Figures 3D–F shows the statistics of the simulated fluorescence distribution. As experimentally observed, in the model the increase of average fluorescence is accompanied by a rapid widening of the distribution, that becomes increasingly asymmetric. Moreover, these processes occur faster in the S condition than in the E condition.

Figure 4B shows the typical dynamics of simulated virions (or fluorescence) distribution. The replication of viral genomes amplifies small differences in growth rate and exponentially stretches the fluorescence repartition. This way, a Gaussian initial growth rate distribution gives rise to highly skewed distributions of fluorescence, thus increasing the relative weight of ‘superspreader’ classes with high viral charge.

In the case of saturating growth, we performed simulations with the same parameters as in condition E, but forcing the average growth rate to be the same as the one measured in the course of the experiment. This leads to a fluorescence distribution wider than in the E condition, and whose moments increase at a progressively slower rate.

However, when cell mortality starts to be important, the model cannot be quantitatively compared to the experimental measures of cell fluorescence, since in the simulations dead cells are immediately removed, while in the culture cells that do not divide seem to accumulate fluorescence before lysing, thus increasing the weight of high fluorescence classes. This or other oversimplifications of the model, that does not take into account any variation in physiological properties at the entry in stationary phase other than the growth rate, may be the reason for the model predicting a quantitatively lower fluorescence than that observed in the experiments in S condition.

### Dynamics of total phage production

**Figure 5:**
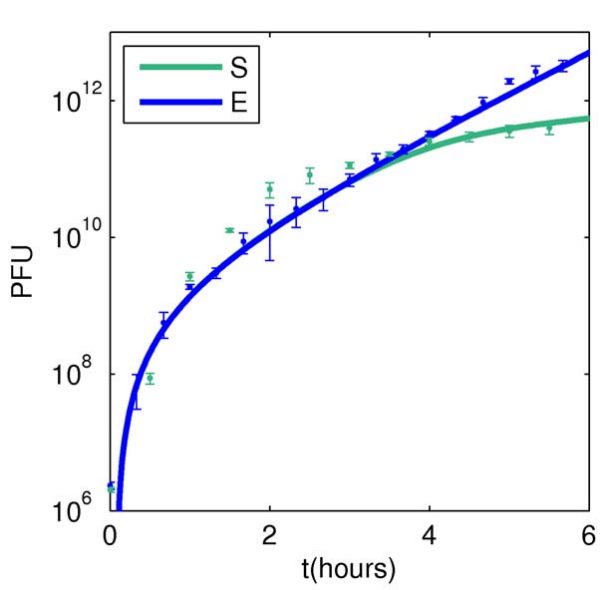
Total virion production. Time evolution of the plaque forming unit (PFU) counts of the whole population in exponential (E, blue symbols) and saturating (S, green symbols) culture conditions following infection by the phage M13yfp. Dots represent the mean +/− the standard error of three independent experiments. In both conditions the increase in PFU is maximal in the first hour following infection. The solid lines represent the total fluorescence calculated by the model (parameters as in Fig. 3) multiplied by a rescaling constant quantifying the ratio (a priori unknown) between measured fluorescence and virions produced per cell.

Total phage production is a quantity typically measured in the course of infections and is currently considered a population-level estimate of virulence. We measured the total virus population in the supernatant (PFU) in the conditions E and S and during 6 hours following infection (Fig. 5). As previously reported [16], the rate of increase in the number of virions released is larger at the beginning of the infection and then attains values comparable to the culture growth rate.

The model points out the processes in the individual-level dynamics and in the distribution kinetics that are important in shaping such a trend in epidemics. Figure 5 shows that the model, whose parameters are constant and obtained by fitting the fluorescence distribution curves, quantitatively predicts the observations in E conditions, and qualitatively matches the experiments in S conditions.

By studying the parameter sensitivity of the model, we can interpret the population-level measures: the initial fast rate of virions production corresponds to the phase of cell-to-cell variability expansion. The cross over to a phase of constant growth rate occurs as the viral concentration within individual cells approaches an equilibrium value, whose magnitude depends on the feedback of virus concentration onto its own production. Such feedback can be due to active regulation of dsDNA replication or to the competition of viruses for cellular resources. In the model, no feedback in virions production is needed for reproducing the infection dynamics, nor is temporal modulation of virions production rate. Instead, cell death seems to be essential in decreasing the virions production rate at the entrance in stationary phase, consistent with the significant percentage of dead cells observed in S growth conditions.

## Discussion

It is commonly observed that differences exist in the individual response to viral infection, from the level of single cells to that of complex organisms endowed with an immune system. This variability in virus production, and in turn in infectiousness, has been mostly attributed to genetic diversity of the host [17] or of its parasite [18]. However, the observation of a correlation between cell size and lysis probability in isogenic bacteria infected by the λ phage [8] has revealed that, at least in the case of virus-bacteria interactions, deterministic causal relationships appear to link the fate of infected cells to physiological parameters. In this work, we have shown that monoclonal cultures of *E. coli* infected by monoclonal viruses display a substantial variability in individual viral production, attaining up to an order of magnitude more variability than other bacterial phenotypic traits. The variation in viral production per cell, has been interpreted for lytic phages as the effect of underlying differences in the burst delay, based on the fact that the autocatalytic deterministic amplification of normally distributed individual parameters can produce log-normal distributions [19]. Our experiments show that a high variability in viral production can be independent of a delay of production, and suggest a different origin of the observed distribution.

In the case of the filamentous M13 phage, we explain the emergence of variability as the outcome of two opposing dynamical processes: the autocatalytic amplification of the virus and its dilution as cells grow. We suggest that a key factor determining the kinetics of viral repartition among cells is the difference in growth rate between the infected bacteria and their parasites: the virus accumulates faster in bacteria growing slower than in those having a faster growth rate. At the level of a population, the same mechanism explains why when bacterial growth slows down, as at the entry in stationary phase, virus production increases.

This mechanism of amplification of small growth rate differences is general and may occur whenever a parasite undergoes autocatalytic replication, that is its production rate is proportional to its cellular concentration. This typically occurs at the very beginning of an infection, and it is such initial stage of an epidemic that the present model aims at describing. Within a short time from the infection, variability in virus production can increase enormously and give rise to highly skewed distributions, as those observed for lytic phages (Fig. 1) and for other viruses [7]. At later stages of the epidemic, several factors may enter into play that could affect the shape of the distribution. First of all, the internal dynamics of virus production: we have chosen here the simplest possible model of density-dependent virus production, a logistic growth. Whenever more complicated regulation mechanisms occur, one may expect more complex scenarii, such as for instance dampened oscillations in the virus concentration or even self-sustained oscillations. These modifications to the model, necessary for a quantitative comparison with specific viral strains, would however not qualitatively affect the initial variability expansion, that is essentially due to the transient exponential growth of viruses at low concentration.

Another factor that is likely to influence the distribution of viruses is the imperfect heritability of growth rate within cell lineages. In the case of *E. coli*, an experimental study has shown that the correlation between the growth rates of sister cells in a lineage is around 0.7 [15]. Taking into account such lineage structure would require analysing models of branching processes that explicitly consider cell division. Such models are expected to converge, for short times, to the simple deterministic limit proposed here.

Besides accurately accounting for the observed distributions of fluorescence and phage productions, the model predicts that cells belonging to classes with growth rate higher than the maximal phage production rate should eventually get rid of the virus, and thus generate a fast-growing subpopulation of ‘healed’ cells, susceptible of being re-infected as soon as the virus stops repressing the production of the F pilus. This scenario is compatible with the observation that after few hours from the infection, non-infected bacteria appear [16].

The proposed mechanism provides a simple explanation for the trade-off between horizontal and vertical transmission commonly observed in parasites that do not induce the death of their host [20], [21]. In our model, such trade-off is a straightforward consequence of growth rate modifications: in harsh conditions for the host, accumulation of viruses in cells favours virions production, i.e. virus horizontal transmission, at the expense of host survival, i.e. virus vertical transmission. In this study, we assume for simplicity that viral replication is independent of host growth rates. This reflects the fact that the initiation of the M13 phage genome replication is determined by phage proteins, and thus independent of the host genome replication machinery. Co-evolution may however have acted so as to maintain viral replication within the range of growth rates attainable by natural populations of the bacterial host. If growth rate differences are present, indeed, more virulent strains would accumulate within the cells and kill their host, and temperate strains would get eventually diluted on longer time scales, thus maintaining the host-parasite system close to critical state.

### BOX: Mathematical Model

We model the population of infected bacteria as composed of a large number of classes, corresponding to different growth rates. These classes can be thought to as being lineages with epigenetic (heritable) differences in doubling time.

Each class is characterized by three variables: the biomass B, the intracellular concentration D of double-stranded viral genome (dsDNA) and the intracellular concentration F of fluorescent proteins. These state variables evolve deterministically based on four parameters: growth rate g of the bacteria, viral DNA duplication rate δ, viral DNA expression rate (or fluorescent protein production rate) τ, carrying capacity K_d_ for viral ds-DNA, and are defined by the following equations:
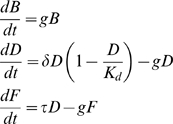
B, D and F are adimentionalised dividing by their unit of measure (not absolute). Each class is initialised with B(0) = 1, D(0) = 1, F(0) = 0, corresponding to a unit of biomass, to a unitary infection (chosen such to maintain the measured ratio D(0)/K_d_ and taken for simplicity identical in all cells), and to an initial absence of fluorescent proteins. The growth rates are assigned according to a given distribution of average g_0_. The distribution of fluorescence is computed taking into account the different biomass of classes with different growth rates and with cells dying stochastically with a probability that is proportional to the fluorescence intensity, up to a threshold value f_thresh_ above which death is certain. Such an assumption is confirmed by the observation that highly fluorescent cells often fail to divide and eventually die.

#### *Parameters choice*

The parameter g_0_ is assigned according to the measured growth rates of infected cultures: for the E condition it is simply the rate of exponential growth, while in the S condition the system is forced by progressively changing g_0_ so as to match the measured values of declining velocity. The growth rate distribution is that measured for non-infected bacteria (Fig. S4c in the SI), and translated so that its average is g_0_ and its standard deviation is maintained to σ = 0.2.

The carrying capacity for the double-stranded DNA (K_d_ = 30) has been taken from independent measures of an average dsDNA content per cell (13).

The parameters δ_LB_ = 1.8 h^−1^ and τ = 30 h^−1^ of the model have been chosen so as to reproduce the experimentally measured shape of the fluorescence distribution and dynamics of its first three moments in E conditions.

The viral DNA duplication rate has to be chosen of the same order of magnitude of the bacterial duplication rate, otherwise either the viral content would explode and the host-parasite couple disappears, or the virus would be diluted out (15).

A fluorescence concentration threshold f_thresh_ = 10^4^, above which the accumulation of fluorescent particles kills the bacterial cells, has been introduced in order to reproduce the large mortality observed in the S condition. This threshold is so high that its effect in the case of exponential growth is minor, in accordance with the fact that a lower number of dead cells is observed in E condition.

#### *Model analysis*

The expansion in the fluorescence concentration reflects the initial exponential growth of the virus. Later, this saturates and a quasi-steady state sets in, where internal concentrations of viruses and fluorescence are stable, but the repartition changes according to the differential demography: classes with higher growth rate tend to be proportionally more represented. The shift of the distribution due to such process is however much slower than the infection dynamics and is almost negligible on the time scale considered here.

Sensitivity analysis has been performed by numerical simulation, starting from an initial Gaussian distribution of mean g_0_ and standard deviation σ. Although the expansion of the initial distribution always occur when parameters are varied, the specific shape of the distribution and the kinetics of its moments depend rather sensitively on the dsDNA duplication rate δ. This reflects the importance of the difference in growth rate between host and parasite, and is confirmed by the fact that similar population dynamics are obtained if the viral growth rate -instead of the bacterial one- is distributed. Increasing δ to values higher than the maximal bacterial growth rate causes the distribution to displace visibly towards higher fluorescence values, before reaching a quasi-steady state, which does not correspond to our observations. Neither does the qualitative behaviour of simulations at low δ values, where after an initial burst, the distribution collapses towards small fluorescence values.

The maximal number of viral dsDNAs sustainable by one cell, K_d_, controls the extent of the distribution expansion: the smaller it is, the narrower the quasi-stationary distribution and the shorter the transient before reaching it.

Finally, the parameter τ measures the fluorescence production rate, but has no quantitative meaning, since fluorescence is measured in relative units.

Modifications in the initial growth rate distribution affect the distribution kinetics in two ways: a decrease/increase in average growth rate is qualitatively equivalent to increasing/decreasing δ, corresponding to the fact that what matters is the growth rate differentials rather than the absolute replication rate of parasite and host.

By changing the variance of the initial growth rate distribution, the main effect is a change in the transient to the quasi-stationary distribution, which is faster for wider distributions.

## Materials and Methods

### Bacterial strains

The *E. coli* strains used were derived from the *E. coli* K12 MG1655 sequenced strain. The MG1655 p2rrnB-cfp strain possessing the reporter p2*rrnB*-*cfp* into the *IntC* gene has been described elsewhere [22]. The MG1655 p2rrnB-cfp F+ strain was constructed by introducing a F plasmid carrying tetracycline resistance by conjugation into the MG1655 p2rrnB-cfp strain.

### Determination of burst size distribution for single cells

A mid–log phase (OD600 between 0.2 and 0.3) *E. coli* MG1655 (or MG1655 F in the case of M13) culture was mixed with phage and incubated at 37°C for 10 minutes, leading to a multiplicity of infection of about 0.4. Infected cells were then diluted in warmed LB to a concentration of 0.3 infected bacteria per 50 µL, and 50 µL of the mixture were distributed in each well of four 96 wells microplates. The microplates were incubated at 37°C during a time equal to the double of the latency period for the lytic phage (latency period of 50 min for R17 and 24 min for T4), and one hour after infection for the phage M13. The number of phage per well was then assayed in triplicates by plaque assay on *E. coli* MG1655 (or MG1655 F) lawn. We obtained between 20 and 33 wells containing phages per microplate. According to a poissonian distribution the proportion of wells containing more than one infected bacteria is expected to be inferior to 4%.

### M13yfp construction

Modifications of the phage genome leading to an even modest perturbation of the phage life cycle can lead to the eventual death of infected cells and generate non-productive infections. For enhancing the probability of obtaining a viable virus, we started from a M13 derivative, the cloning vector M13mp19 (New England Biolabs, Ipswitch, Massachusset, United States), which possesses the LacZα gene in its origin of replication. To construct the reporter p2*rrnB*-*yfp*, sequence encoding the fluorescent protein YFP-v+ was cloned upstream of the promoter p2 of the *rrnB* operon (p2*rrnB*: from nucleotides 152 to 94 relative to the translation start of the *rrsB* gene). M13yfp was constructed by introducing the p2*rrnB*-*yfp* construct into the polycloning site of M13mp19, between the KpnI and FspI sites.

In order to quantify cell-cell differences in fluorescence due to variations in the activity of the promoter, we incorporated the distinguishable cyan (cfp) allele of the yfp gene in the the chromosome of the bacterial host (see bacterial strains section) and under the same promoter p2*rrnB*. A very weak correlation between the fluorescence levels in yfp and in cfp (R2 = 0,033 for a linear regression), indicates that the observed variability is not explained by differences in the *rrnB* promoter activity.

### Fluorescence distribution assays

#### *Growth conditions*

*E. coli* MG1655 p2rrnB-cfp F cultures growing exponentially in LB (OD600 between 0.2 and 0.3) were mixed with phages at a multiplicity of infection (m.o.i.) of 20 and incubated at 37°C for 15 minutes without agitation. These conditions ensure that 90% of the bacterial population is infected at the time of dilution. M13yfp infected cells were then diluted 20 times in 20 mL of LB (DIFCO, Becton Dickinson) and incubated in a water bath at 37°C with shaking. The culture was then let enter the stationary phase (case S, saturating growth), re-diluted every hour with fresh warm LB medium (case E, exponential growth), or diluted every hour in 2-hours conditioned media supplemented with yeast extract.

#### *Single-cell imaging*

Samples were taken at 20 or 30 minutes intervals. In order to obtain a monolayer of cells, samples of growing cultures of M13yfp infected cells were concentrated by centrifugation (1.5 min at maximum speed in a bench centrifuge) and spread on LBagarose (Qbiogene) supplemented with chloramphenicol (Sigma, 30 µg/ml) and propidium iodide (Molecular Probes, 0.5 µg/ml). A series of images of cell monolayer was taken with a CoolSNAP HQ (Princeton Instruments, Trenton, New Jersey, USA) at 100× magnification by an automated microscope (Zeiss 200M; Zeiss, Jena, Germany), in phase contrast and in fluorescence at wavelength 514 nm (YFP), 540nm (propidium iodide) and 420nm (CFP) during 1s exposure time. Excitation light was limited to 50% of the output of the 100-W Hg vapor lamp.

#### *Image analysis*

Images were treated with the Metamorph software (Universal Imaging, Downingtown, Pennsylvania, USA). Image analysis procedure identified cells and then quantified their mean fluorescent intensities with YFP, CFP and IP filter sets. We analysed more than 10,000 cells at each time point. Fluorescent background of the LBagarose media was subtracted from each value of fluorescence. Cells were considered dead if the IP fluorescence level was above 1.5 times the medium value of the IP fluorescence of all cells.

### Model simulations and statistical analysis

The model simulations and statistical analysis were performed with MATLAB (Mathworks, Natick, MA). The same code has been used for the statistical analysis of both the experimental data and the simulated distributions.

## Supporting Information

Figure S1 Scheme of M13 life cycle. Upon entering the cell, the phage genome is duplicated. Subsequently, transcription, replication, and generation of single-strand genomes occur. Phage proteins assemble around single-stranded genomes to produce virions that are subsequently extruded from the cell. The intracellular dynamics of phage replication in individual cells was tracked by means of a fluorescent gene reporter introduced in the genome of the filamentous phage M13mp19, a derivative of phage M13.
(0.06 MB TIF)

Figure S2 Effects of yfp insertion on growth rates of the phage and its host. A Effect of yfp insertion on the host. As previously reported, infection reduces the culture growth rate, and the same bacterial growth rate reduction was obtained with the WT M13 phage, the phage M13mp19 possessing a polycloning site in its genome and the YFP encoding phage M13 Yfp. This indicates that the cost of infection does not increase with the size of the phage. The same bacterial population has been infected at time 0 at a multiplicity of infection of 0.1. B effect of gene insertion on phage doubling time (mean +/− standard deviation). The phage doubling time is the time necessary for the number of free virion to double, and is calculated from the exponential increase in PFU measured from 0.5 to 2 hours after infection.
(0.02 MB TIF)

Figure S3 Relation between the number of phage produced and phage-encoded fluorescence. Number of total PFU counts versus total fluorescence of the culture. The measures have been made between 3 and 6 hours after the beginning of the infection in 166 distinct M13Yfp infected bacterial cultures. Solid line: exponential regression, R2 = 0.88. During conditions of sustained growth of the infected cells, the number of phage particles produced is proportional to the total yfp fluorescence of the culture, indicating a correlation between yfp intensity and number of virions per cell. The use of fluorescence as a measure of virions production is also validated by the comparison of single-cell fluorescence distribution and PFU counts on single-cells (Figure 2 c).
(0.01 MB TIF)

Figure S4 Distribution of physiological bacterial parameters. A and B: At each time point, the distribution of CFP fluorescence intensity, which reflects chromosomal expression of the reporter gene, show only relatively low cell-cell variation (A) and follows a distribution whose characteristics are stable with time (B). This is consistent with our construction that places CFP under a constitutive promoter. The relative error is about 10%. C: Distribution of the log2 transformation of the growth rate of non-infected bacterial cells. Growth rates are obtained be time-lapse microscopy of cells plated on agar in nutrient-rich conditions as described in (Stewart, 2005). The bacterial growth rate follows an almost normal distribution of average 2.1 hours-1 and standard deviation 0.2 hours-1. This is used, after rescaling to the measured growth rate of infected cells, as the initial condition for the model simulations.
(6.42 MB TIF)
