# Supplementary figures and images for: Emergence of Variability in Isogenic Escherichia coli Populations Infected by a Filamentous Virus
Source: PLoS One. 2010 Jul 27;5(7):e11823. doi: 10.1371/journal.pone.0011823 (PMC2910729; doi:10.1371/journal.pone.0011823)

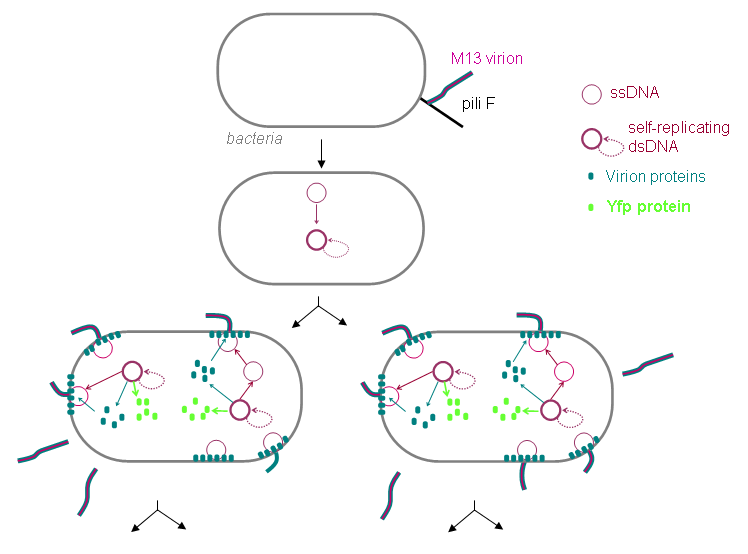

Supplement: Figure S1 — Scheme of M13 life cycle. Upon entering the cell, the phage genome is duplicated. Subsequently, transcription, replication, and generation of single-strand genomes occur. Phage proteins assemble around single-stranded genomes to produce virions that are subsequently extruded from the cell. The intracellular dynamics of phage replication in individual cells was tracked by means of a fluorescent gene reporter introduced in the genome of the filamentous phage M13mp19, a derivative of phage M13. (0.06 MB TIF) [file pone.0011823.s001.tif]

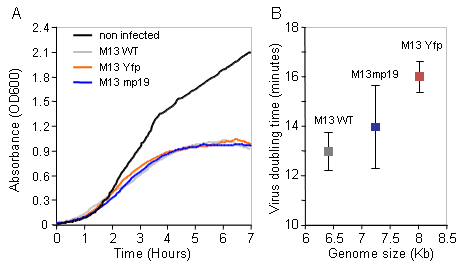

Supplement: Figure S2 — Effects of yfp insertion on growth rates of the phage and its host. A Effect of yfp insertion on the host. As previously reported, infection reduces the culture growth rate, and the same bacterial growth rate reduction was obtained with the WT M13 phage, the phage M13mp19 possessing a polycloning site in its genome and the YFP encoding phage M13 Yfp. This indicates that the cost of infection does not increase with the size of the phage. The same bacterial population has been infected at time 0 at a multiplicity of infection of 0.1. B effect of gene insertion on phage doubling time (mean +/− standard deviation). The phage doubling time is the time necessary for the number of free virion to double, and is calculated from the exponential increase in PFU measured from 0.5 to 2 hours after infection. (0.02 MB TIF) [file pone.0011823.s002.tif]

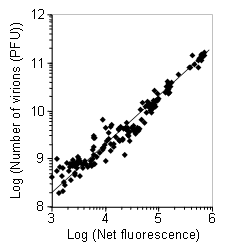

Supplement: Figure S3 — Relation between the number of phage produced and phage-encoded fluorescence. Number of total PFU counts versus total fluorescence of the culture. The measures have been made between 3 and 6 hours after the beginning of the infection in 166 distinct M13Yfp infected bacterial cultures. Solid line: exponential regression, R2 = 0.88. During conditions of sustained growth of the infected cells, the number of phage particles produced is proportional to the total yfp fluorescence of the culture, indicating a correlation between yfp intensity and number of virions per cell. The use of fluorescence as a measure of virions production is also validated by the comparison of single-cell fluorescence distribution and PFU counts on single-cells (Figure 2 c). (0.01 MB TIF) [file pone.0011823.s003.tif]

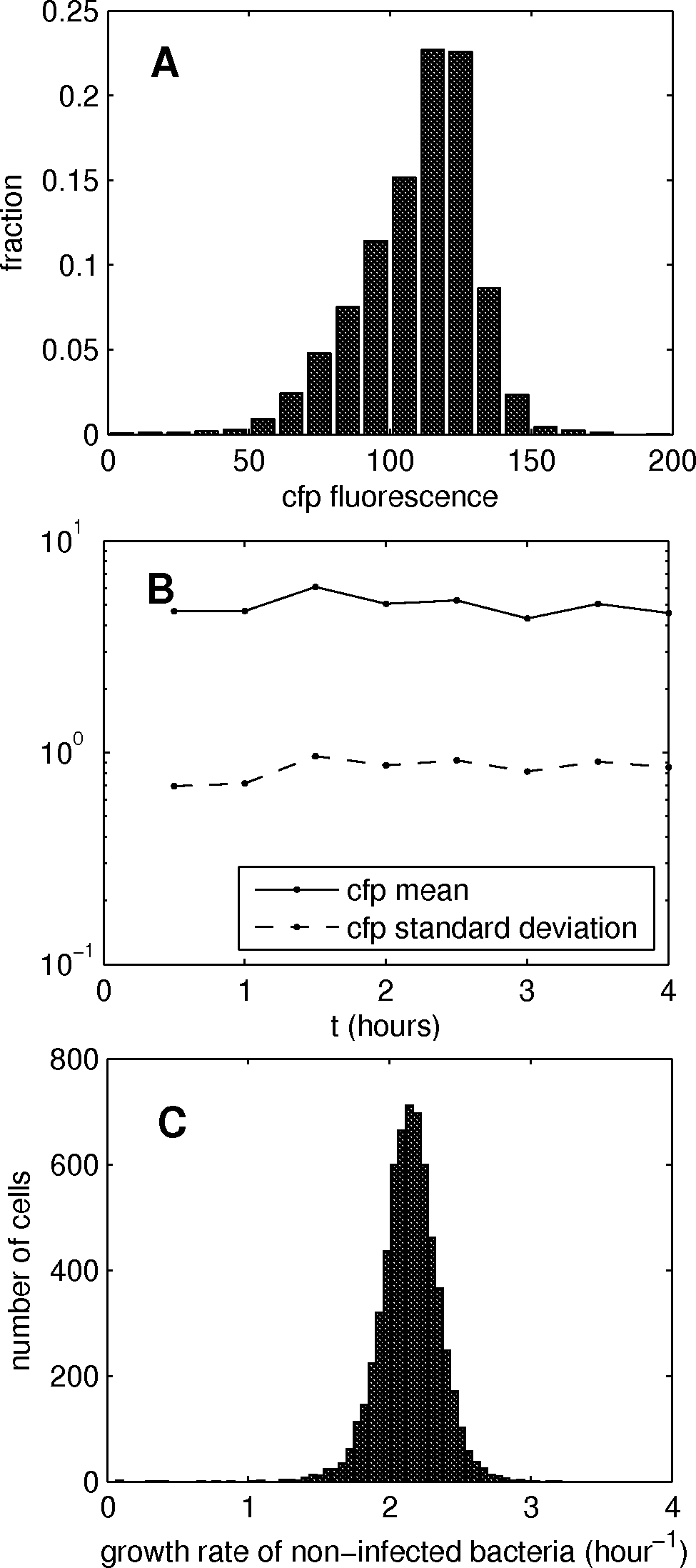

Supplement: Figure S4 — Distribution of physiological bacterial parameters. A and B: At each time point, the distribution of CFP fluorescence intensity, which reflects chromosomal expression of the reporter gene, show only relatively low cell-cell variation (A) and follows a distribution whose characteristics are stable with time (B). This is consistent with our construction that places CFP under a constitutive promoter. The relative error is about 10%. C: Distribution of the log2 transformation of the growth rate of non-infected bacterial cells. Growth rates are obtained be time-lapse microscopy of cells plated on agar in nutrient-rich conditions as described in (Stewart, 2005). The bacterial growth rate follows an almost normal distribution of average 2.1 hours-1 and standard deviation 0.2 hours-1. This is used, after rescaling to the measured growth rate of infected cells, as the initial condition for the model simulations. (6.42 MB TIF) [file pone.0011823.s004.tif]
